# Reaching key populations through key venues: Insights from the Jamaica HIV Prevention Program

**DOI:** 10.1371/journal.pone.0206962

**Published:** 2018-11-26

**Authors:** Sharon S. Weir, J. Peter Figueroa, Marion Scott, Lovette Byfield, Carol Jones Cooper, Marcia C. Hobbs

**Affiliations:** 1 Department of Epidemiology, University of North Carolina, Chapel Hill, North Carolina, United States of America; 2 Department of Community Health and Psychiatry, University of the West Indies, Mona, Kingston, Jamaica; 3 Ministry of Health, Kingston, Jamaica; 4 Epidemiology and Research Training Unit, Kingston, Jamaica; 5 Department of Medicine, University of North Carolina, North Carolina, United States of America; Johns Hopkins University, UNITED STATES

## Abstract

**Introduction:**

HIV prevention strategies often include outreach to female sex workers at social venues identified as places where people meet new sexual partners. Patrons and staff at these venues may include female sex workers, their clients, as well as others who have high rates of new sexual partnerships. Few studies have compared HIV/STI among venue-based and general populations, across types of venues, or by sub-group of the venue population. Program planners often assume that the prevalence of infection is highest among female sex workers and considerably lower among other people at these venues, but there are few empiric studies assessing the prevalence of infection by sex worker status and type of venue.

**Methods:**

In 2011, we used the PLACE method to identify public venues where people meet new sexual partners across Jamaica. The study team visited all venues with reported sex work as well as a 10% random sample of other venues and subsequently interviewed and tested a probability sample of 991 venue patrons and workers for HIV and other STI.

**Results:**

Community informants identified 1207 venues. All venues where sex work was reported (735 venues) and a random sample of the remainder (134 of 472) were selected for onsite visits. Of these, 585 were found and operational. At a stratified random sample of venues, survey teams interviewed and tested 717 women and 274 men. 394 women reported recent sex work and 211 of these women reported soliciting clients on the street. Women exchanging sex for money were more likely to be infected with HIV (5.4% vs 1.0%; OR = 5.6, 95% CI = 1.8,17.3) or syphilis (11.7% vs. 5.8%, OR = 2.2, 95% CI = 1.7,4,0) than other women, but not significantly more likely to be infected with gonorrhea (8.4% vs 7.8%; OR = 1.1,95% CI = 0.6,1.9), chlamydia (16.2% vs 21.6%;OR = 0.7,95% CI = 0.5,1.0) or trichomoniasis (23.0% vs 17.0%, OR = 1.5,95% CI = 0.9,2.2). Women at venues were more likely to report sex work and multiple partners than women interviewed in a 2008 national population-based household survey commissioned by the Ministry of Health.

**Conclusions:**

In Jamaica, although the highest HIV prevalence was among street-based sex workers, the risk of HIV and STI extends to men and women at high risk venues, even those who do not self-identify as sex workers. Findings confirm the appropriateness of outreach to all men and women at these venues.

## Introduction

The World Health Organization recommends focused HIV programming for men who have sex with men, people who inject drugs, sex workers, people in closed settings, and transgender people [1]. These groups are more likely to acquire and transmit infection and less likely to access services. A major challenge in service delivery is how to reach these populations who may not trust service delivery providers or have access to public health care.

Vertical approaches, such as drop-in centers operated by non-governmental organizations, can reach key populations. These programs, however, are often small-scale, provide limited services, and can have problems linking clients to services without “outing” the person as a member of a key population.

An alternative approach supplements public health clinic-based services with outreach to high risk public venues such as bars and night clubs, with the expectation of reaching sex workers and others at increased risk in the environment where risk behavior is encouraged. Outreach may be more convenient for participants and may reach those who do not perceive their risk or do not visit clinics or drop-in centers due to fear, lack of resources, or other reason. Venue-based HIV prevention strategies range from providing condoms in hotels [2] to comprehensive approaches [3]. Criticisms of venue-based strategies relative to vertical approaches include the lack of one-stop provision of services, lack of a key-population friendly environment, intrusion into a social environment, and failure to reach those who do not visit venues.

Here we assess the relevance of a venue-based outreach strategy in Jamaica, a country with a mixed epidemic profile [4, 5]. Adult HIV prevalence is 1.7%. An estimated 28% of the approximately 30,300 adults living with HIV [6] are unaware of their infection. National population-based cross-sectional surveys suggest a fairly stable risk profile (over the period of 2004 to 2012) with 14% of women and 47% of men reporting multiple sexual partners in the past 12 months [7]. Previous surveys indicate high HIV prevalence among MSM (31.4%) and transgender women (52.9%) [8]; and high prevalence among female sex workers (4.9%) [9].

## Methods

The Priorities for Local AIDS Control Efforts (PLACE) method [10] systematically identifies, characterizes, and maps venues where people most likely to acquire and transmit HIV can be reached by programs [6]. The strategy is to reach people with the highest rates of new sexual partnerships at places where are were most likely to meet new partners.

Since 2002, the Ministry of Health in Jamaica has conducted outreach to high-risk venues [11]. The Jamaica PLACE strategy, first implemented in the parishes of St James [11, 12] and Kingston & St Andrew [13, 14] [6], has been extended to all parishes. Implementation focused on urban areas and “vulnerable communities” identified based on a rough algorithm that took into account population density, poverty, literacy, known sex work, violence, and reported HIV/AIDS cases. The authors led the development and implementation of the PLACE strategy in Jamaica, most since its initial implementation.

The conceptual model underlying the program was the proximate determinants framework[15] which describes pathways from underlying determinants to HIV transmission via the proximate determinants of increased transmissibility, exposure, and susceptibility. We further developed the initial framework by expanding the categories of underlying determinants to include vulnerabilities, adverse life events, and disinhibiting behaviors.

### PLACE program

The PLACE program in Jamaica included three elements:

1. Venue mapping every 3–5 years to identify, map and characterize all public venues in the selected communities where people meet new sexual partners.
2. Bio-behavioral surveys of patrons and workers every 3–5 years.
3. Ongoing programmatic response by trained outreach workers.

#### Venue mapping

Trained interviewers across all regions of Jamaica asked community informants (such as street vendors and taxi drivers) to identify places where people meet new sexual partners. Interviewers asked for verbal informed consent. No personal identifiers were requested. For each venue identified, the respondent provided its name, address, type, and whether sex work occurs onsite. The target number of informants was 1200, but interviewers continued until no new places were named. The results were compiled into a comprehensive venue list.

Next, interviewers visited all night clubs, exotic dance clubs, massage parlors and venues with reported onsite sex work as well as a 10% random sample of other venues where sex work was not reported. Interviewers asked willing knowledgeable respondents at the venue, called “venue informants” about the venue, including whether men come to the venue looking for sex workers.

#### Bio-behavioral surveys of patrons and workers

A cross-sectional sample of approximately 500 female workers, 300 female patrons, and 300 male patrons was interviewed at a sample of the venues that were visited. The sample size was allocated across the four regions based on population size (Table 1).

**Table 1:** Target Number of Interviews by Region and type of venue, PLACE, Jamaica 2011.

	Target Number of Interviews with Patrons and Workers		
Health Region	Female Workers	Female Patrons	Male Patrons
South East	275	165	165
North East	75	45	45
Western	125	75	75
Southern	25	15	15
Total	500	300	300

The study design was a stratified two-stage cluster sample. Venues were stratified into four strata, with the first three comprised of venues initially identified as those with reported sex work and the last one including venues without reported sex work:

1. Sex Worker Street Venues
2. Exotic dance clubs and massage parlors;
3. All others with reported sex work.
4. Venues without reported sex work.

The first stage of sampling selected the venues to be visited. As described above, all venues in the first three strata were visited as well as a 10% random sample of the fourth stratum. In the second stage of sampling, where the objective was to select venues for onsite interview with patrons and workers, a random sample of venues in each stratum was selected, with oversampling of stratum 1 and 2.

Within selected venues, participants were randomly selected with the number interviewed proportional to the number onsite. Sampling weights reflect the probability that the venue was selected for a venue visit (Stage 1) and for the onsite survey and test (Stage 2). We analyzed survey data using proc surveyfreq in SAS, using the stratum as the stratum variable, the venue identification number as the cluster, and the sampling weight as the weight.

Informed consent was requested. Participants age 16 years or older were asked to initialize a consent form and agree to the face-to-face onsite structured interview and tests. Workers were interviewed before the venue was busy. Surveys asked about sexual behavior, STI symptoms, exposure to health programs, and knowledge of HIV. For the most sensitive questions, the respondent privately recorded answers on an answer sheet, which was subsequently sealed in an envelope. No identifying information was obtained by interviewers.

In 2011, testing included rapid tests for HIV (*Determine*/ *Unigold)*, a combined urine test for gonorrhea and chlamydia (Gen-Probe APTIMA Combo2), a rapid test for syphilis (SD *BIOLINE*) confirmed by T*reponema pallidum* hemagglutination assay, and a urine test for trichomoniasis (Gen-Probe APTIMA TV ASR). Since anal swabs were not collected, results do not cover rectal gonorrhea or chlamydia. A phone card (US $6) was provided for calling for test results. HIV testing was conducted at the same time as the survey by Ministry of Health personnel using standard approved Ministry of Health protocols. A code linked test and survey results. HIV test results were provided at the time of the survey. Urine samples were collected and tested later. Efforts were made to inform people of all test results.

The authors developed survey protocols in collaboration with the National HIV Program and key stakeholders including Jamaica AIDS Support, USAID, CDC and the Jamaica Red Cross. The Institutional Review Board of the University of North Carolina and the Ethical Review Committee of the Jamaica Ministry of Health approved the protocols.

#### Ongoing programmatic response by trained outreach workers

Outreach workers provided peer education, HIV testing, education regarding risk reduction and condom use, and personal risk assessments to address gaps. They were trained in behavior change communication theory, the facts of HIV transmission, motivational interviewing, human sexuality, values clarification, self-efficacy, stigma and discrimination reduction, risk reduction conversations, voluntary counseling and testing, and skills building in condom negotiation and condom use.

### Assessment of venue-based approach: Objectives and methods

Four issues were addressed to assess the venue-based approach:

1. Comparison with General Population. To assess whether PLACE reached persons at increased risk, we compared three key behavioral risk indicators (age at first sex, multiple sexual partnerships, and sex work) from PLACE with indicators from the 2008 Jamaica National KAP Survey. We restricted the PLACE sample to women of the same age as women in the KAP survey. The national KAP survey is a survey of a national probability sample of household residents commissioned by the Government of Jamaica and implemented in 2008 by Hope Enterprises.
2. Yield by Venue Type. To determine whether the efficiency of reaching those at highest risk could be improved by targeting a certain type of venue, we compared the risk profile (based on the proximate determinants [15] of HIV transmission) by venue category. We compared prevalence of HIV/STI, risk behaviors, reported sex work, and underlying risk determinants for men and women by type of venue (streets, clubs and massage parlors, and other venues).
3. Sex Worker Risk. To assess the extent to which sex workers were more at risk than other women, we compared the prevalence of HIV and STI among women by sex work statu
4. Access to services. To estimate the proportion of respondents tested for HIV and contacted through outreach.

#### Statistical analysis

Statistical analysis was performed using SAS/STAT version 14.3. We calculated point estimates and confidence intervals for PLACE data with proc surveyfreq in SAS, using venue group as the survey stratum variable, the venue as cluster, and the sampling weight as the weight. Similarly, for estimates from the National Knowledge, Attitudes and Behavior (KAPB) survey, point estimates and confidence intervals controlled for parish as the survey stratum and survey enumeration unit as the cluster. We calculated standard Wald 95% confidence limits with proc surveyfreq in SAS using variance estimates based on the sample design. To assess differences by type of venue, we calculated Rao-Scott chi-square statistics using SAS surveyfreq separately for men and women taking into account survey stratum, clustering by venue and sampling weights. Due to the large number of comparisons, we set the p value for determination of significance at p < .01. Due to sparse sample size for men and women from venues without sex work (n = 56), we combined the data from stratum 3 and 4. Confidence intervals are provided in the supplemental materials. Differences in means were assessed using proc surveymeans, accounting for the sampling design, and setting the value for significance at p<0.01.

## Results

In 2011, community informants identified 1207 unique venues and events. These were compiled into a venue list. 869 were selected for a venue visit: 735 with onsite sex work and 134 chosen randomly. 585 were successfully visited (485 sex work sites and 100 randomly selected sites). Of those not completed: 76 had missing or incomplete questionnaires; 54 sites refused; 60 sites were permanently closed; 54 were temporarily closed; 34 were not feasible to visit; and 6 could not be located.

**Fig 1:**
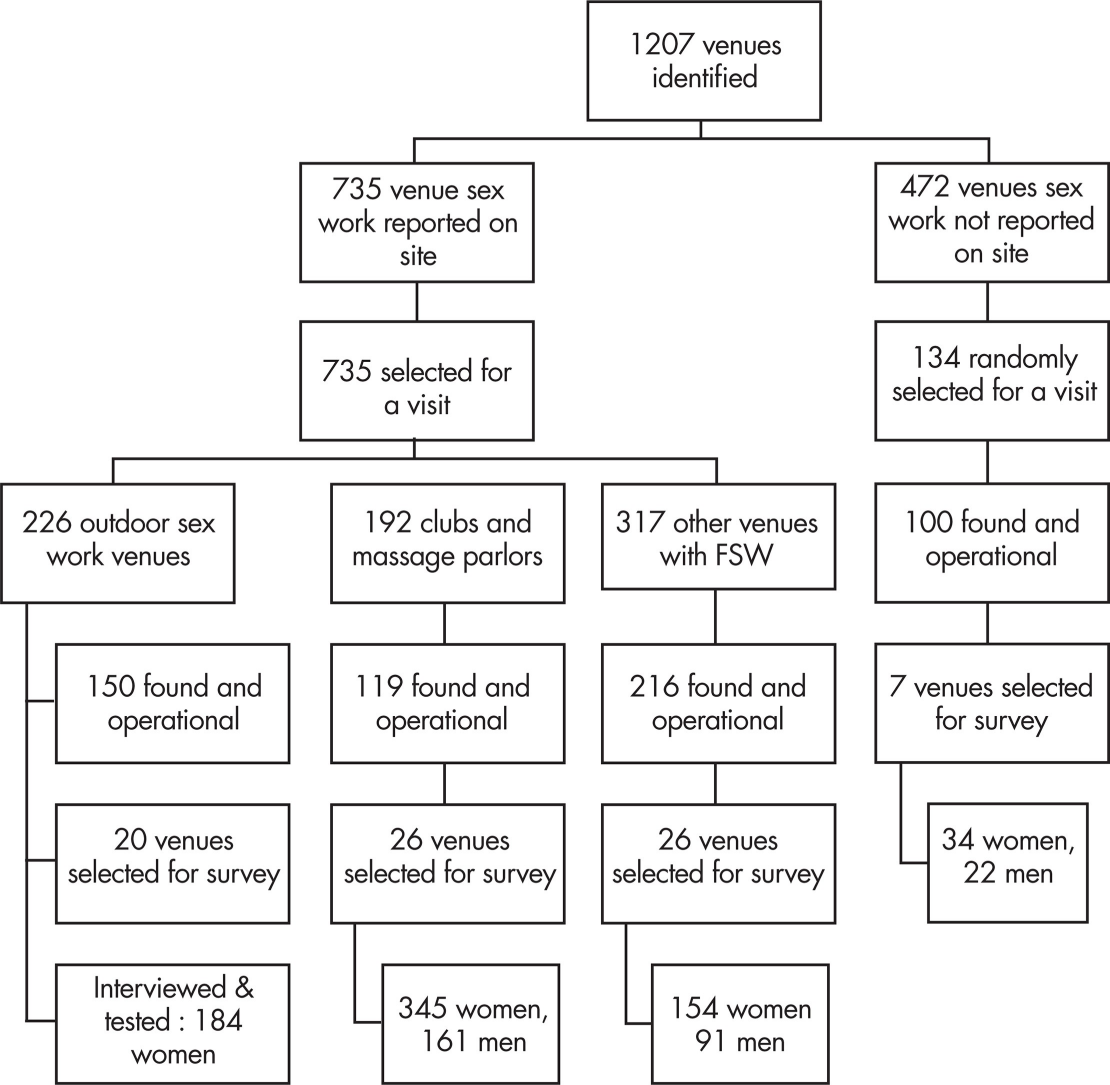
Sampling flow chart by venue group and sample size.

Worker and patron surveys were conducted between September and December 2011. 464 female workers, 253 female patrons, and 274 male patrons were interviewed and tested. 368 women reported exchanging sex for money in the past three months (Fig 1).

### Comparison with the general population

Women at venues were twice as likely (24.0% vs 11.1%) to report sex before age 15 compared with women in the general population; three times as likely (47% vs 15%) to report more than one sexual partner in the past year; and over ten times as likely to report receiving cash for sex (48% vs 1.8%). More men at PLACE venues (76%) reported multiple sexual partners in the past year than men in the general population (52%) (Table 2).

**Table 2:** Indicators from national population based household (HH) Survey (2008) in Jamaica compared with PLACE sex workers and non-sex workers.

	—————————-Women———————				——-Men——	
	HH Survey	—————PLACE—————-			HH Survey	PLACE
	Women	All	Sex Work	No Sex Work	Men	All
Aged 16–24 N	396	282	135	147	378	89
Reports sex <age 15%	11.1	23.6	32.0	17.2	41.8	56.5
*95% CI*	*8*.*0*,*14*.*2*	*18*.*9*, *28*.*3*	*24*.*9*, *39*.*0*	*11*.*3*, *23*.*1*	*35*.*9*,*47*.*7*	*44*.*1*, *68*.*9*
Aged 16–49 N	854	697	362	335	827	256
>1 partner past year %	15	71.8	98.7	47.3	52.0	76.1
*95% CI*	*12*.*8*,*17*.*2*	*67*.*1*, *76*.*6*	*97*.*3*,*100*.*0*	*41*.*2*,*53*.*4*	*47*.*7*,*56*.*3*	*70*.*7*, *81*.*6*
Sex work past year %	1.8	47.8	100.0	—	—	—
*95% CI*	*0*.*9*, *2*.*7*	*41*.*3*, *54*.*2*				

### Importance of venue type: Infection, determinants, vulnerability

We found significant differences in HIV and syphilis prevalence, sexual behavior, condom use, vulnerability factors, reported adverse life events, and disinhibiting behaviors among women by venue type (Table 3). There were almost no significant differences by venue type for men.

**Table 3:** Characteristics of female workers (FW), female patrons (FP) and male patrons (MP) at streets, clubs, and other venues in Jamaica 2011.

Sex	———————Female————————					——-Male—-		
Venue Type	Street	Clubs		Other		Clubs	Other	
Group	Worker	Worker	Patron	Worker	Patron	Patron	Patron	*p < .01
Number of Respondents (N)	184	205	140	75	113	161	113	
**HIV and STI Outcomes**								
HIV Infection %	7.1	2.9	0.7	1.2	0.8	0.6	0.0	F*
Gonorrhea %	9.8	9.7	6.8	7.8	5.5	5.2	2.9	
Chlamydia %	16.3	20.0	22.7	25.2	15.7	22.2	19.6	
Syphilis %	15.2	4.9	5.9	6.7	5.9	4.4	4.4	F*
Trichomonas %	25.0	19.9	18.2	13.3	18.3	3.4	7.6	
% Infected with HIV, Gonorrhea, Chlamydia, Trichomonas or Syphilis	43.5	33.2	36.4	34.2	31.0	22.4	23.9	
Infected with >1 STI %	2.2	2.4	0.7	1.2	0.0	0.0	0.0	
**Proximate Determinants**								
• **Of Increased Exposure to HIV**								
Aged < 16 at first sex %	53.9	50.5	43.8	29.6	33.9	73.0	68.9	F*
Partners last 4 weeks (mean)	30.1	8.8	2.3	1.0	1.0	2.8	1.7	F*
New partners last 4 weeks (mean)	19.6	5.3	1.0	0.2	0.3	1.4	0.9	
Mean N Partners last year	207.4	54.7	8.4	1.6	2.1	10.3	9.2	
2+ partners past 4 weeks %	87.0	69.8	36.4	12.8	11.6	44.1	36.1	F*
New partner in past year %	85.9	73.7	50.7	24.9	27.1	57.1	57.9	F*
Believes partner has other partners %	40.8	43.9	52.9	55.9	45.0	36.0	16.0	M*
Anal sex in the past year %	29.3	18.0	17.9	5.5	12.8	19.3	15.3	F*
Injected non-prescription drug past 6 months %	0.5	2.0	3.6	0.0	0.8	0.6	0.8	
• **Of Transmission if Exposed**								
Reported anal sex past year and no condom last anal sex %	16.8	10.3	9.9	3.4	11.6	11.0	7.6	
No condom last vaginal sex %	17.4	36.0	52.7	55.1	57.9	49.7	59.7	F*
2+ partners past 4 weeks and no condom at last sex %	10.9	19.4	16.8	9.0	9.9	18.1	17.7	F*
• **Low Risk**								
No new partners past year, partner has no other partners, used condom last vaginal sex %	1.1	4.1	8.4	9.0	14.0	4.6	7.6	F*
• **Underlying Determinants**								
• **General**								
Mean Age (years) %	30.1	26.5	26.2	31.7	30.1	30.5	33.5	
Have any children %	79.1	72.4	66.9	75.4	63.4	55.0	60.6	
Have main male sex partner %	67.2	68.8	78.6	85.2	75.0	0.7	4.5	
Have main female sex partner %	7.7	14.7	10.9	8.2	4.2	73.3	72.6	
Married/living with partner %	28.8	30.2	31.4	35.1	27.7	25.5	25.9	
• **Vulnerabilities**								
Younger than 25 years %	31.0	41.5	54.3	25.8	38.7	32.3	31.5	
Less than secondary school education %	9.2	3.4	1.4	0.0	6.7	2.5	5.0	F*
Unemployed %	45.4	18.5	42.9	4.6	38.2	23.1	19.5	F*
Low socio-economic status %	26.1	26.3	17.1	29.6	16.7	25.5	26.6	
Low literacy %	7.6	1.5	0.7	0.0	0.8	1.2	7.3	F*
• **Adverse Life Events**								
Ever spent night in jail %	34.8	25.4	19.3	8.1	13.0	34.8	30.5	F*
Ever Raped %	21.2	22.4	19.3	11.3	6.9	4.3	2.9	F*
Ever slept outdoors because homeless %	16.8	15.1	8.6	2.3	5.3	18.0	12.9	F*
• **Disinhibiting Behaviors**								
Reported exchanging sex for money in the past 3 months %	90.2	72.2	30.7	2.3	7.1	12.4	5.2	F*
Ever paid cash for sex %	21.4	10.8	10.8	2.3	4.4	46.6	34.4	F*
Used crack or cocaine past 6 months %	1.6	1.0	0.0	0.0	0.0	0.6	2.1	
Daily alcohol consumption past 4 weeks %	20.7	38.0	23.6	15.7	10.2	29.2	23.7	F*
Visit venue 4+ times per week %	47.3	68.8	20.7	84.3	34.6	22.4	35.5	
Visited or plans to visit two venues tonight %	35.9	22.0	40.0	6.4	36.0	37.9	36.5	

FW = Female Worker; FP = Female Patron; MP-Male Patron. In the farright column, F* indicates a significant difference (p<0.01) in the indicator for women across venue type. M* indicates a significant difference for men.

#### Street venues

Female workers on the streets had the highest prevalence of HIV (7.1%), syphilis (15.2%), trichomoniasis (25.0%) and any infection (43.5%) (Table 3). The mean number of sexual partnerships in the past four weeks was highest among women interviewed at street sites (30.1). Most of the street-based workers (90.2%) reported sex work. Street-based women were least likely to have completed secondary school, most likely to report unemployment, and most likely to have difficulty reading. We estimate that there are approximately 2100 women working on the streets in Jamaica (an average of 14 per venue according to informants at 150 locations).

#### Clubs and massage parlors

We estimated that 1,500 women work in clubs, massage parlors and other sex work venues in Jamaica (an average of 13 per venue according to venue informants at 119 venues). Most engaged in sex work (72.2%). Estimated STI prevalence included: chlamydia (20.0%), trichomonas (19.9%) and gonorrhea (9.7%). Compared with street-based women, these workers were younger, more likely to drink alcohol daily (38% vs 21%), have fewer sexual partners (8.8 vs 30 in the past 4 weeks), more likely not to use condoms (36% vs 17%), and equally likely to report rape (21–22%).

We estimated that about 3000 women visit clubs, massage parlors and other sex work venues on a peak night as patrons. These women have a similar burden of STI as the workers in these venues even though they are less likely to report sex work (31%). Patrons were just as likely as workers, however, to engage in unprotected anal sex (9.9% vs 10.3%), almost as likely to have multiple partners in the past 4 weeks and not use a condom (16.8% vs 19.4%) and more likely to have not used a condom at last sex (52.7% vs. 36%).

We estimated approximately 2500 men visit clubs and massage parlors at peak times in Jamaica. These presumably include male partners of the women at these women. The most common STIs among men are chlamydia (22.2%) gonorrhea (5.2%) and syphilis (4.4%). Almost half (44%) report having two or more sexual partners in the past 4 weeks. Almost 20% reported anal sex in the past year and 11% reported not using a condom during the most recent anal sex. Like women at these venues, half of the men reported not using a condom at last vaginal sex. Less than 1% of male club patrons reported that their main sexual partner was male. 4.3% reported being raped and over a third had spent a night in jail. Almost half (46%) reported paying cash for sex and 22% reported visiting the venue four or more times per week.

#### Other venues

These venues include bars (other than night clubs), restaurants, parks, malls, and street dances and are generally considered lower risk. Female workers at these venues, however, were just as likely to be infected with chlamydia (25%) as women at clubs and more likely than women on the street (16%). The average number of sexual partnerships in the past 4 weeks (1.0) and past year (2.1) among female workers at these other venues was lower than among women working at clubs, but condom use was also lower.

24% of men at other venues had evidence of an STI: chlamydia (20%), trichomoniasis (7%), syphilis (4.4%), gonorrhea (3%) and HIV (0%). Men patronizing these sites were older than the women and almost all were heterosexual. Many reported spending a night in jail (31%) or sleeping outside due to homelessness (13%). One quarter reported daily alcohol use. Over 30% reported ever paying cash for sex. The mean number of sexual partners was 1.7 in the past four weeks and 9.2 in the past 12 months. 57% reported a new sexual partner in the past 12 months.

### Female sex worker risk

When asked, a third of venue informants reported that men come to the site to buy sex from women. This proportion was highest at street sites and clubs but was not uncommon at the other venues where 16% reported that men come to buy sex.

Women exchanging sex for money were more likely to be infected with HIV (5.4% vs 1.0%; OR = 5.6, 95% CI = 1.8,17.3) or syphilis (11.7% vs. 5.8%, OR = 2.2, 95% CI = 1.7,4,0) than other women, but not significantly more likely to be infected with gonorrhea (8.4% vs 7.8%), chlamydia (16.2% vs 21.6%) or trichomoniasis (23.0% vs 17.0%,). Sex workers on the street and in clubs were most likely to have any STI, but the differences were not large—at least 30% of the other women had at least one STI.

### Access to services

Over 90% of the women working on streets, in clubs or massage parlors had been tested for HIV, most within the past 12 months (Table 4). Most had talked with an outreach worker about their risk of getting HIV and testing. Men were less likely to have been tested in the past 12 months or to have talked with an outreach worker about testing.

**Table 4:** HIV testing and risk reduction contact with outreach workers among workers and patrons at venues.

Sex	—————Female———————————					——Male——		
Venue Type	Street	Clubs		Other		Clubs	Other	
	Worker	Worker	Patron	Worker	Patron	Patron	Patron	*p < .01
Number	184	205	140	75	113	161	113	
	%	%	%	%	%	%	%	
Never tested for HIV	8.2	6.3	22.1	31.0	27.5	43.5	40.2	F*
Tested in past year	79.9	76.0	51.4.	36.5	39.4	32.3	40.0	F*
Talked with outreach worker about HIV risk	76.8	62.3	17.5	31.6	21.6	15.9	14.3	F*
Talked with outreach worker about HIV test	79.2	64.7	19.3	30.3	24.2	17.5	14.3	F*
Have seen risk card	58.4	58.4	32.6	38.9	50.2	35.2	35.4	

*p<0.01.

## Discussion

The findings provide evidence of the advantages of venue-based outreach, particularly for women, and provide insight into how to enhance the effectiveness of a venue-based outreach strategy through venue targeting.

A few points warrant discussion. First, the venue-based population has a higher risk profile than the general population. Women at PLACE venues report more sexual partnerships, a younger age at first sex, and more sex work. Even women at PLACE venues who denied sex work were three times more likely to report more than one partner in the past year as women in the general population (47% vs 15%). The men at PLACE venues are at higher risk of HIV and other STI than men in the general population; 68% of them report two or more sexual partners in the past year and over 30% reported ever paying cash for sex. The comparison is somewhat compromised because the household survey did not include HIV/STI testing; the time and place of implementation differed somewhat; and the interview location (household vs social venue) differed. However, even within the general population survey, the fact that those who report visiting social venues have a higher risk profile than those who do not (data not shown) supports the interpretation that the venue-going population is more at risk than the general population.

Second, the findings confirm that street-based women are at highest risk for acquiring and transmitting HIV and syphilis. Their HIV prevalence was highest (7.1%) and 43% percent had evidence of HIV, gonorrhea, chlamydia, trichomonas or syphilis infection. Onward transmission is likely: they reported, on average, 30 sex partners in the past 4 weeks, of whom 20 were new partners. 90% reported sex work in the past 3 months. These findings are not surprising. Street-based sex work is often the most challenging, dangerous, and risky for women and poses the most challenges for outreach. The Jamaica program has appropriately focused on street-based sex work for many years. An impressive 80% of these women reported an HIV test within the past year and talking with an outreach worker.

Third, we expected that each STI infection would vary by venue type, but the prevalence of gonorrhea, chlamydia and trichomonas did not vary by venue type for men or women. At least 30% of women and 22% of men had evidence of at least one infection. Future analyses will explore the association between the HIV/STI outcomes and injecting drug use, unprotected sex and anal sex.

Fourth, surprisingly, although we expected sex workers to have a higher prevalence of STI than other women, the prevalence of any infection was similar (33% vs 36%). In some cases, non-sex workers were more likely to be infected with an STI: for example, at other social venues, non-sex workers were more likely to be infected with gonorrhea than sex workers (7.1% vs 3.2%). One interpretation is that women at these other venues, who may exchange sex for goods or services, do not-self-identify as sex workers, perceive themselves at lower risk, have multiple partners, but are less likely to use condoms. According to the Jamaica national sexual behavior survey in 2008, among women aged 15–49 sexually active in the past year, 1.8% reported exchanging sex for cash in the past 12 months but 17.2% reported receiving money for expenses such as food, clothing, entertainment or bus fare in exchange for sex [7]. The UNFPA recommends defining sex workers [16] to include people who engage in part-time sex work or who exchange sex for goods or services. Although some of these women are clearly at increased risk in Jamaica, it would not be appropriate to apply this definition of sex work in Jamaica because it conflates sex work and transactional relationships and would label nearly a fifth of women in the country as sex workers [9].

Fifth, the findings demonstrate the current reach of a venue-based strategy in Jamaica: almost 80% of those interviewed reported a recent HIV test, 80% had talked with an outreach worker about their risk of getting HIV; and 80% talked with an outreach worker about HIV testing. 79% of female workers on the street and 75% of female workers at clubs and massage parlors reported being HIV tested in the past year. HIV prevalence trends among sex workers declined from 12% in 1989 [8, 11] to 4% in 2011, suggesting that the approach is effective as well as feasible.

Sixth, the findings support outreach to men. The prevalence of chlamydia among male patrons was approximately 20%. Half of men did not use a condom at last vaginal sex. Social vulnerabilities among men were common: 21% were unemployed, over 30% had spent a night in jail; and 12% of male patrons at clubs and 7% of male patrons at other venues reported sex work in the past three months.

Seventh, venue outreach to reduce unprotected anal sex may be appropriate. Strategies to reduce unprotected anal sex among men who have sex with men have been underway in Jamaica for years, however, the program has not used a venue-based approach for these men. Site informants reported that MSM patronized 13% of the venues. Unprotected anal sex also occurs among women. One quarter (29%) of women working on the street, 17.9% of women working in clubs and massage parlors and 15% of female and male patrons reported anal sex in the past year, often without a condom.

In summary, we found that a venue-based approach can reach people at high risk of STI, including those who do not self-identify as a member of one of WHO’s five key populations. STI prevalence varied by type of venue. The study confirmed that street sex workers operate in the most vulnerable environments, are most likely to self-identify as sex workers, have the highest number of sexual partners and are more likely to be infected with HIV. Women in other settings, particularly those with multiple partners who do not self-identify as sex workers, however, remain at risk. They may tend to discount their behavioral risk and fail to consistently use condoms. The Jamaica PLACE program has proven itself capable of reaching people at these venues to promote HIV testing and risk reduction and attributes the reduction in HIV prevalence among female sex workers in Jamaica to these efforts. Future challenges will be to extend prevention outreach to more men and women at these venues.

## Supporting information

S1 Table Confidence limits for Tables 3 and 4. (PDF)

S1 File (SAS7BDAT)
