# Supplementary material for: Reaching key populations through key venues: Insights from the Jamaica HIV Prevention Program
Source: PLoS One. 2018 Nov 26;13(11):e0206962. doi: 10.1371/journal.pone.0206962 (PMC6261031; doi:10.1371/journal.pone.0206962)
Supplement: S1 Table — (PDF) [file pone.0206962.s001.pdf]

Table of Group by HIV12

Group	HIV12	Frequency	Weighted Frequency	Row Percent	95% Confidence Limits for Row Percent	
Street:Female Workers	Not infected	171	1283	92.9	89.1	96.7
	Infected	13	98	7.1	3.3	10.9
	Total	184	1380	100.0		
Clubs:Female Workers	Not infected	199	911	97.1	94.9	99.3
	Infected	6	27	2.9	0.7	5.1
	Total	205	938	100.0		
Clubs Female Patrons	Not infected	139	636	99.3	97.9	100.0
	Infected	1	5	0.7	0.0	2.1
	Total	140	641	100.0		
Other Sites:Female Workers	Not infected	74	704	98.8	96.5	100.0
	Infected	1	8	1.2	0.0	3.5
	Total	75	713	100.0		
Other Sites:Female Patrons	Not infected	112	1044	99.2	97.7	100.0
	Infected	1	8	0.8	0.0	2.3
	Total	113	1052	100.0		
Clubs Male Patrons	Not infected	160	732	99.4	98.2	100.0
	Infected	1	5	0.6	0.0	1.9
	Total	161	737	100.0		
Other Sites:Male Patrons: run	Not infected	113	1070	100.0	100.0	100.0
	Infected	0	.	.	.	.
	Total	113	1070	100.0		
Total	Not infected	968	6381			
	Infected	23	151			
	Total	991	6531			

Table of Group by ng12

Group	ng12	Frequency	Weighted Frequency	Row Percent	95% Confidence Limits for Row Percent	
Street:Female Workers	Not infected	166	1245	90.2	85.0	95.4
	Infected	18	135	9.8	4.6	15.0
	Total	184	1380	100.0		
Clubs:Female Workers	Not infected	177	810	90.3	84.0	96.6
	Infected	19	87	9.7	3.4	16.0
	Total	196	897	100.0		
Clubs Female Patrons	Not infected	123	563	93.2	88.5	97.9
	Infected	9	41	6.8	2.1	11.5
	Total	132	604	100.0		
Other Sites:Female Workers	Not infected	69	657	92.2	85.6	98.7
	Infected	6	56	7.8	1.3	14.4
	Total	75	713	100.0		
Other Sites:Female Patrons	Not infected	106	994	94.5	90.1	98.9
	Infected	7	58	5.5	1.1	9.9
	Total	113	1052	100.0		
Clubs Male Patrons	Not infected	145	664	94.8	91.3	98.2

	Infected	8	37	5.2	1.8	8.7
	Total	153	700	100.0		
Other Sites:Male Patrons: run	Not infected	109	1025	97.1	93.8	100.0
	Infected	3	31	2.9	0.0	6.2
	Total	112	1056	100.0		
Total	Not infected	895	5958			
	Infected	70	445			
	Total	965	6403			

Frequency Missing = 26

Table of Group by ct12

Group	ct12	Frequency	Weighted Frequency	Row Percent	95% Confidence Limits for Row Percent	
Street:Female Workers	Not infected	154	1155	83.7	77.6	89.8
	Infected	30	225	16.3	10.2	22.4
	Total	184	1380	100.0		
Clubs:Female Workers	Not infected	156	714	80.0	73.9	86.1
	Infected	39	179	20.0	13.9	26.1
	Total	195	893	100.0		
Clubs Female Patrons	Not infected	102	467	77.3	69.7	84.8
	Infected	30	137	22.7	15.2	30.3
	Total	132	604	100.0		
Other Sites:Female Workers	Not infected	57	533	74.8	65.1	84.6
	Infected	18	179	25.2	15.4	34.9
	Total	75	713	100.0		
Other Sites:Female Patrons	Not infected	96	887	84.3	78.6	90.0
	Infected	17	165	15.7	10.0	21.4
	Total	113	1052	100.0		
Clubs Male Patrons	Not infected	119	545	77.8	69.8	85.7
	Infected	34	156	22.2	14.3	30.2
	Total	153	700	100.0		
Other Sites:Male Patrons: run	Not infected	90	849	80.4	71.7	89.2
	Infected	22	207	19.6	10.8	28.3
	Total	112	1056	100.0		
Total	Not infected	774	5150			
	Infected	190	1248			
	Total	964	6398			

Frequency Missing = 27

Table of Group by SYPH12

Group	SYPH12	Frequency	Weighted Frequency	Row Percent	95% Confidence Limits for Row Percent	
Street:Female Workers	Not infected	156	1170	84.8	78.2	91.3
	Infected	28	210	15.2	8.7	21.8
	Total	184	1380	100.0		
Clubs:Female Workers	Not infected	194	888	95.1	92.1	98.1

	Infected	10	46	4.9	1.9	7.9
	Total	204	934	100.0		
Clubs Female Patrons	Not infected	127	581	94.1	90.3	97.9
	Infected	8	37	5.9	2.1	9.7
	Total	135	618	100.0		
Other Sites:Female Workers	Not infected	70	665	93.3	86.7	99.9
	Infected	5	48	6.7	0.1	13.3
	Total	75	713	100.0		
Other Sites:Female Patrons	Not infected	107	991	94.1	90.2	98.1
	Infected	6	62	5.9	1.9	9.8
	Total	113	1052	100.0		
Clubs Male Patrons	Not infected	151	691	95.6	92.5	98.7
	Infected	7	32	4.4	1.3	7.5
	Total	158	723	100.0		
Other Sites:Male Patrons: run	Not infected	108	1023	95.6	91.8	99.3
	Infected	5	48	4.4	0.7	8.2
	Total	113	1070	100.0		
Total	Not infected	913	6009			
	Infected	69	481			
	Total	982	6490			

Frequency Missing = 9

Table of Group by tv12

Group	tv12	Frequency	Weighted Frequency	Row Percent	95% Confidence Limits for Row Percent	
Street:Female Workers	Not infected	138	1035	75.0	66.0	84.0
	Infected	46	345	25.0	16.0	34.0
	Total	184	1380	100.0		
Clubs:Female Workers	Not infected	157	719	80.1	74.3	85.9
	Infected	39	179	19.9	14.1	25.7
	Total	196	897	100.0		
Clubs Female Patrons	Not infected	108	494	81.8	75.0	88.6
	Infected	24	110	18.2	11.4	25.0
	Total	132	604	100.0		
Other Sites:Female Workers	Not infected	65	618	86.7	78.2	95.1
	Infected	10	95	13.3	4.9	21.8
	Total	75	713	100.0		
Other Sites:Female Patrons	Not infected	92	860	81.7	74.8	88.7
	Infected	21	192	18.3	11.3	25.2
	Total	113	1052	100.0		
Clubs Male Patrons	Not infected	148	677	96.7	93.3	100.0
	Infected	5	23	3.3	0.0	6.7
	Total	153	700	100.0		
Other Sites:Male Patrons: run	Not infected	103	975	92.4	87.3	97.4
	Infected	9	81	7.6	2.6	12.7
	Total	112	1056	100.0		
Total	Not infected	811	5378			
	Infected	154	1024			
	Total	965	6403			

Frequency Missing = 26

Table of Group by anysti

Group	anysti	Frequency	Weighted Frequency	Row Percent	95% Confidence Limits for Row Percent	
Street:Female Workers	Not infected	104	780	56.5	47.9	65.1
	Infected	80	600	43.5	34.9	52.1
	Total	184	1380	100.0		
Clubs:Female Workers	Not infected	137	627	66.8	58.5	75.2
	Infected	68	311	33.2	24.8	41.5
	Total	205	938	100.0		
Clubs Female Patrons	Not infected	89	407	63.6	54.3	72.8
	Infected	51	233	36.4	27.2	45.7
	Total	140	641	100.0		
Other Sites:Female Workers	Not infected	50	469	65.8	54.8	76.9
	Infected	25	244	34.2	23.1	45.2
	Total	75	713	100.0		
Other Sites:Female Patrons	Not infected	78	726	69.0	60.0	78.0
	Infected	35	327	31.0	22.0	40.0
	Total	113	1052	100.0		
Clubs Male Patrons	Not infected	125	572	77.6	69.6	85.7
	Infected	36	165	22.4	14.3	30.4
	Total	161	737	100.0		
Other Sites:Male Patrons: run	Not infected	85	814	76.0	66.6	85.5
	Infected	28	257	24.0	14.5	33.4
	Total	113	1070	100.0		
Total	Not infected	668	4395			
	Infected	323	2136			
	Total	991	6531			

Table of Group by coinfected

Group	coinfected	Frequency	Weighted Frequency	Row Percent	95% Confidence Limits for Row Percent	
Street:Female Workers	Not infected	180	1350	97.8	96.0	99.6
	Infected	4	30	2.2	0.4	4.0
	Total	184	1380	100.0		
Clubs:Female Workers	Not infected	200	915	97.6	95.5	99.6
	Infected	5	23	2.4	0.4	4.5
	Total	205	938	100.0		
Clubs Female Patrons	Not infected	139	636	99.3	97.9	100.0
	Infected	1	5	0.7	0.0	2.1
	Total	140	641	100.0		
Other Sites:Female Workers	Not infected	74	704	98.8	96.5	100.0
	Infected	1	8	1.2	0.0	3.5
	Total	75	713	100.0		
Other Sites:Female Patrons	Not infected	113	1052	100.0	100.0	100.0
	Infected	0	.	.	.	.
	Total	113	1052	100.0		
Clubs Male Patrons	Not infected	161	737	100.0	100.0	100.0
	Infected	0	.	.	.	.
	Total	161	737	100.0		
Other Sites:Male Patrons: run	Not infected	113	1070	100.0	100.0	100.0
	Infected	0	.	.	.	.

	Total	113	1070	100.0		
Total	Not infected	980	6466			
	Infected	11	66			
	Total	991	6531			

Table of Group by youngsex

Group	youngsex	Frequency	Weighted Frequency	Row Percent	95% Confidence Limits for Row Percent	
Street:Female Workers	No	83	623	46.1	38.2	54.1
	Yes	97	728	53.9	45.9	61.8
	Total	180	1350	100.0		
Clubs:Female Workers	No	101	462	49.5	42.8	56.3
	Yes	103	471	50.5	43.7	57.2
	Total	204	934	100.0		
Clubs Female Patrons	No	77	352	56.2	48.5	63.9
	Yes	60	275	43.8	36.1	51.5
	Total	137	627	100.0		
Other Sites:Female Workers	No	51	495	70.3	60.2	80.5
	Yes	23	209	29.7	19.5	39.8
	Total	74	704	100.0		
Other Sites:Female Patrons	No	73	684	66.1	56.8	75.3
	Yes	38	352	33.9	24.7	43.2
	Total	111	1036	100.0		
Clubs Male Patrons	No	40	183	27.0	19.1	35.0
	Yes	108	494	73.0	65.0	80.9
	Total	148	677	100.0		
Other Sites:Male Patrons: run	No	34	312	31.1	21.9	40.3
	Yes	71	691	68.9	59.7	78.1
	Total	105	1004	100.0		
Total	No	459	3112			
	Yes	500	3220			
	Total	959	6332			

Frequency Missing = 32

Table of Group by twoplus4

Group	twoplus4	Frequency	Weighted Frequency	Row Percent	95% Confidence Limits for Row Percent	
Street:Female Workers	No	24	180	13.0	4.2	21.9
	Yes	160	1200	87.0	78.1	95.8
	Total	184	1380	100.0		
Clubs:Female Workers	No	62	284	30.2	22.9	37.6
	Yes	143	655	69.8	62.4	77.1
	Total	205	938	100.0		
Clubs Female Patrons	No	89	407	63.6	53.7	73.5
	Yes	51	233	36.4	26.5	46.3
	Total	140	641	100.0		
Other Sites:Female Workers	No	64	621	87.2	78.2	96.1
	Yes	11	91	12.8	3.9	21.8
	Total	75	713	100.0		
Other Sites:Female Patrons	No	99	930	88.4	82.9	93.8
	Yes	14	122	11.6	6.2	17.1
	Total	113	1052	100.0		

Clubs Male Patrons	No	90	412	55.9	48.2	63.6
	Yes	71	325	44.1	36.4	51.8
	Total	161	737	100.0		
Other Sites:Male Patrons: run	No	73	684	63.9	53.9	74.0
	Yes	40	386	36.1	26.0	46.1
	Total	113	1070	100.0		
Total	No	501	3519			
	Yes	490	3013			
	Total	991	6531			

Table of Group by anynew

Group	anynew	Frequency	Weighted Frequency	Row Percent	95% Confidence Limits for Row Percent	
Street:Female Workers	No	26	195	14.1	7.8	20.5
	Yes	158	1185	85.9	79.5	92.2
	Total	184	1380	100.0		
Clubs:Female Workers	No	54	247	26.3	19.6	33.0
	Yes	151	691	73.7	67.0	80.4
	Total	205	938	100.0		
Clubs Female Patrons	No	69	316	49.3	42.4	56.2
	Yes	71	325	50.7	43.8	57.6
	Total	140	641	100.0		
Other Sites:Female Workers	No	55	535	75.0	65.7	84.3
	Yes	20	178	25.0	15.7	34.3
	Total	75	713	100.0		
Other Sites:Female Patrons	No	80	766	72.8	63.2	82.4
	Yes	33	286	27.2	17.6	36.8
	Total	113	1052	100.0		
Clubs Male Patrons	No	69	316	42.9	36.4	49.3
	Yes	92	421	57.1	50.7	63.6
	Total	161	737	100.0		
Other Sites:Male Patrons: run	No	47	450	42.1	34.6	49.6
	Yes	66	620	57.9	50.4	65.4
	Total	113	1070	100.0		
Total	No	400	2825			
	Yes	591	3706			
	Total	991	6531			

Table of Group by cheats

Group	cheats	Frequency	Weighted Frequency	Row Percent	95% Confidence Limits for Row Percent	
Street:Female Workers	No	109	818	59.2	50.5	68.0
	Yes	75	563	40.8	32.0	49.5
	Total	184	1380	100.0		
Clubs:Female Workers	No	115	526	56.1	49.1	63.1
	Yes	90	412	43.9	36.9	50.9
	Total	205	938	100.0		
Clubs Female Patrons	No	66	302	47.1	39.8	54.5
	Yes	74	339	52.9	45.5	60.2
	Total	140	641	100.0		
Other Sites:Female Workers	No	35	315	44.2	33.9	54.4

	Yes	40	398	55.8	45.6	66.1
	Total	75	713	100.0		
Other Sites:Female Patrons	No	64	580	55.1	46.7	63.4
	Yes	49	473	44.9	36.6	53.3
	Total	113	1052	100.0		
Clubs Male Patrons	No	103	471	64.0	55.1	72.9
	Yes	58	265	36.0	27.1	44.9
	Total	161	737	100.0		
Other Sites:Male Patrons: run	No	93	898	83.9	76.0	91.8
	Yes	20	172	16.1	8.2	24.0
	Total	113	1070	100.0		
Total	No	585	3910			
	Yes	406	2622			
	Total	991	6531			

Table of Group by anal

Group	anal	Frequency	Weighted Frequency	Row Percent	95% Confidence Limits for Row Percent	
Street:Female Workers	No	130	975	70.7	64.5	76.8
	Yes	54	405	29.3	23.2	35.5
	Total	184	1380	100.0		
Clubs:Female Workers	No	168	769	82.0	74.7	89.2
	Yes	37	169	18.0	10.8	25.3
	Total	205	938	100.0		
Clubs Female Patrons	No	115	526	82.1	76.2	88.1
	Yes	25	114	17.9	11.9	23.8
	Total	140	641	100.0		
Other Sites:Female Workers	No	71	674	94.5	89.2	99.8
	Yes	4	39	5.5	0.2	10.8
	Total	75	713	100.0		
Other Sites:Female Patrons	No	99	918	87.2	78.4	96.1
	Yes	14	134	12.8	3.9	21.6
	Total	113	1052	100.0		
Clubs Male Patrons	No	130	595	80.7	73.7	87.8
	Yes	31	142	19.3	12.2	26.3
	Total	161	737	100.0		
Other Sites:Male Patrons: run	No	97	907	84.8	77.3	92.3
	Yes	16	163	15.2	7.7	22.7
	Total	113	1070	100.0		
Total	No	810	5364			
	Yes	181	1167			
	Total	991	6531			

Table of Group by inject

Group	inject	Frequency	Weighted Frequency	Row Percent	95% Confidence Limits for Row Percent	
Street:Female Workers	No	183	1373	99.5	98.4	100.0
	Yes	1	8	0.5	0.0	1.6
	Total	184	1380	100.0		
Clubs:Female Workers	No	201	920	98.0	96.2	99.9
	Yes	4	18	2.0	0.1	3.8
	Total	205	938	100.0		

Clubs Female Patrons	No	135	618	96.4	92.3	100.0
	Yes	5	23	3.6	0.0	7.7
	Total	140	641	100.0		
Other Sites:Female Workers	No	75	713	100.0	100.0	100.0
	Yes	0	.	.	.	.
	Total	75	713	100.0		
Other Sites:Female Patrons	No	112	1044	99.2	97.7	100.0
	Yes	1	8	0.8	0.0	2.3
	Total	113	1052	100.0		
Clubs Male Patrons	No	160	732	99.4	98.2	100.0
	Yes	1	5	0.6	0.0	1.8
	Total	161	737	100.0		
Other Sites:Male Patrons: run	No	112	1062	99.2	97.7	100.0
	Yes	1	8	0.8	0.0	2.3
	Total	113	1070	100.0		
Total	No	978	6461			
	Yes	13	70			
	Total	991	6531			

Table of Group by uai

Group	uai	Frequency	Weighted Frequency	Row Percent	95% Confidence Limits for Row Percent	
Street:Female Workers	No	152	1140	82.6	76.6	88.7
	Yes	32	240	17.4	11.3	23.4
	Total	184	1380	100.0		
Clubs:Female Workers	No	185	847	90.2	85.3	95.2
	Yes	20	92	9.8	4.8	14.7
	Total	205	938	100.0		
Clubs Female Patrons	No	125	572	89.3	84.8	93.8
	Yes	15	69	10.7	6.2	15.2
	Total	140	641	100.0		
Other Sites:Female Workers	No	73	696	97.7	94.4	100.0
	Yes	2	17	2.3	0.0	5.6
	Total	75	713	100.0		
Other Sites:Female Patrons	No	101	941	89.4	81.5	97.3
	Yes	12	112	10.6	2.7	18.5
	Total	113	1052	100.0		
Clubs Male Patrons	No	144	659	89.4	84.4	94.5
	Yes	17	78	10.6	5.5	15.6
	Total	161	737	100.0		
Other Sites:Male Patrons: run	No	104	990	92.5	87.7	97.2
	Yes	9	81	7.5	2.8	12.3
	Total	113	1070	100.0		
Total	No	884	5844			
	Yes	107	687			
	Total	991	6531			

Table of Group by uvi

Group	uvi	Frequency	Weighted Frequency	Row Percent	95% Confidence Limits for Row Percent	
Street:Female Workers	No	152	1140	82.6	75.2	90.0

	Yes	32	240	17.4	10.0	24.8
	Total	184	1380	100.0		
Clubs:Female Workers	No	134	613	65.4	59.4	71.4
	Yes	71	325	34.6	28.6	40.6
	Total	205	938	100.0		
Clubs Female Patrons	No	67	307	47.9	37.9	57.8
	Yes	73	334	52.1	42.2	62.1
	Total	140	641	100.0		
Other Sites:Female Workers	No	28	268	37.7	25.7	49.6
	Yes	47	444	62.3	50.4	74.3
	Total	75	713	100.0		
Other Sites:Female Patrons	No	47	438	41.6	34.7	48.6
	Yes	66	614	58.4	51.4	65.3
	Total	113	1052	100.0		
Clubs Male Patrons	No	82	375	50.9	41.2	60.7
	Yes	79	362	49.1	39.3	58.8
	Total	161	737	100.0		
Other Sites:Male Patrons: run	No	44	419	39.2	29.2	49.2
	Yes	69	651	60.8	50.8	70.8
	Total	113	1070	100.0		
Total	No	554	3561			
	Yes	437	2970			
	Total	991	6531			

Table of Group by twoplusnocondom

Group	twoplusnocondom	Frequency	Weighted Frequency	Row Percent	95% Confidence Limits for Row Percent	
Street:Female Workers	No	164	1230	89.1	84.3	94.0
	Yes	20	150	10.9	6.0	15.7
	Total	184	1380	100.0		
Clubs:Female Workers	No	167	764	81.5	76.3	86.6
	Yes	38	174	18.5	13.4	23.7
	Total	205	938	100.0		
Clubs Female Patrons	No	116	531	82.9	75.4	90.3
	Yes	24	110	17.1	9.7	24.6
	Total	140	641	100.0		
Other Sites:Female Workers	No	68	655	91.8	86.1	97.6
	Yes	7	58	8.2	2.4	13.9
	Total	75	713	100.0		
Other Sites:Female Patrons	No	103	969	92.1	87.0	97.2
	Yes	10	83	7.9	2.8	13.0
	Total	113	1052	100.0		
Clubs Male Patrons	No	133	609	82.6	76.6	88.6
	Yes	28	128	17.4	11.4	23.4
	Total	161	737	100.0		
Other Sites:Male Patrons: run	No	92	866	80.9	74.2	87.7
	Yes	21	204	19.1	12.3	25.8
	Total	113	1070	100.0		
Total	No	843	5624			
	Yes	148	908			
	Total	991	6531			

Table of Group by lowrisk

Group	lowrisk	Frequency	Weighted Frequency	Row Percent	95% Confidence Limits for Row Percent	
Street:Female Workers	No	182	1365	98.9	97.3	100.0
	Yes	2	15	1.1	0.0	2.7
	Total	184	1380	100.0		
Clubs:Female Workers	No	197	902	96.1	93.9	98.3
	Yes	8	37	3.9	1.7	6.1
	Total	205	938	100.0		
Clubs Female Patrons	No	129	590	92.1	87.7	96.6
	Yes	11	50	7.9	3.4	12.3
	Total	140	641	100.0		
Other Sites:Female Workers	No	67	640	89.8	83.4	96.3
	Yes	8	72	10.2	3.7	16.6
	Total	75	713	100.0		
Other Sites:Female Patrons	No	96	905	86.0	79.5	92.5
	Yes	17	147	14.0	7.5	20.5
	Total	113	1052	100.0		
Clubs Male Patrons	No	153	700	95.0	91.8	98.3
	Yes	8	37	5.0	1.7	8.2
	Total	161	737	100.0		
Other Sites:Male Patrons: run	No	105	992	92.7	88.2	97.1
	Yes	8	78	7.3	2.9	11.8
	Total	113	1070	100.0		
Total	No	929	6095			
	Yes	62	437			
	Total	991	6531			

Table of Group by anychildren

Group	anychildren	Frequency	Weighted Frequency	Row Percent	95% Confidence Limits for Row Percent	
Street:Female Workers	No	32	240	20.9	14.2	27.7
	Yes	121	908	79.1	72.3	85.8
	Total	153	1148	100.0		
Clubs:Female Workers	No	56	256	27.6	19.5	35.6
	Yes	147	673	72.4	64.4	80.5
	Total	203	929	100.0		
Clubs Female Patrons	No	46	211	33.1	24.6	41.6
	Yes	93	426	66.9	58.4	75.4
	Total	139	636	100.0		
Other Sites:Female Workers	No	19	176	24.7	14.0	35.4
	Yes	56	537	75.3	64.6	86.0
	Total	75	713	100.0		
Other Sites:Female Patrons	No	41	382	36.6	24.5	48.8
	Yes	71	662	63.4	51.2	75.5
	Total	112	1044	100.0		
Clubs Male Patrons	No	72	330	45.0	35.8	54.2
	Yes	88	403	55.0	45.8	64.2
	Total	160	732	100.0		
Other Sites:Male Patrons: run	No	45	422	39.4	28.6	50.2
	Yes	68	649	60.6	49.8	71.4
	Total	113	1070	100.0		

Total	No	311	2016			
	Yes	644	4256			
	Total	955	6272			

Frequency Missing = 36

Table of Group by Q23A

Group	Q23A	Frequency	Weighted Frequency	Row Percent	95% Confidence Limits for Row Percent	
Street:Female Workers	No	60	450	32.8	25.0	40.6
	Yes	123	923	67.2	59.4	75.0
	Total	183	1373	100.0		
Clubs:Female Workers	No	64	293	31.2	23.2	39.2
	Yes	141	645	68.8	60.8	76.8
	Total	205	938	100.0		
Clubs Female Patrons	No	30	137	21.4	12.5	30.3
	Yes	110	503	78.6	69.7	87.5
	Total	140	641	100.0		
Other Sites:Female Workers	No	12	106	14.8	6.5	23.2
	Yes	63	607	85.2	76.8	93.5
	Total	75	713	100.0		
Other Sites:Female Patrons	No	31	264	25.0	17.0	33.1
	Yes	82	789	75.0	66.9	83.0
	Total	113	1052	100.0		
Clubs Male Patrons	No	152	696	99.3	98.1	100.0
	Yes	1	5	0.7	0.0	1.9
	Total	153	700	100.0		
Other Sites:Male Patrons: run	No	102	961	95.5	90.6	100.0
	Yes	4	45	4.5	0.0	9.4
	Total	106	1006	100.0		
Total	No	451	2906			
	Yes	524	3517			
	Total	975	6423			

Frequency Missing = 16

Table of Group by Q23B

Group	Q23B	Frequency	Weighted Frequency	Row Percent	95% Confidence Limits for Row Percent	
Street:Female Workers	No	155	1163	92.3	88.5	96.0
	Yes	13	98	7.7	4.0	11.5
	Total	168	1260	100.0		
Clubs:Female Workers	No	168	769	85.3	78.6	91.9
	Yes	29	133	14.7	8.1	21.4
	Total	197	902	100.0		
Clubs Female Patrons	No	114	522	89.1	80.6	97.5
	Yes	14	64	10.9	2.5	19.4
	Total	128	586	100.0		
Other Sites:Female Workers	No	65	624	91.8	84.7	98.9
	Yes	6	56	8.2	1.1	15.3
	Total	71	680	100.0		
Other Sites:Female Patrons	No	102	949	95.8	91.4	100.0
	Yes	5	42	4.2	0.0	8.6
	Total	107	991	100.0		

Clubs Male Patrons	No	43	197	26.7	18.5	35.0
	Yes	118	540	73.3	65.0	81.5
	Total	161	737	100.0		
Other Sites:Male Patrons: run	No	30	285	27.4	17.3	37.4
	Yes	81	757	72.6	62.6	82.7
	Total	111	1042	100.0		
Total	No	677	4508			
	Yes	266	1688			
	Total	943	6196			

Frequency Missing = 48

Table of Group by marliv

Group	marliv	Frequency	Weighted Frequency	Row Percent	95% Confidence Limits for Row Percent	
Street:Female Workers	No	131	983	71.2	65.1	77.3
	Yes	53	398	28.8	22.7	34.9
	Total	184	1380	100.0		
Clubs:Female Workers	No	143	655	69.8	63.4	76.1
	Yes	62	284	30.2	23.9	36.6
	Total	205	938	100.0		
Clubs Female Patrons	No	96	439	68.6	60.1	77.1
	Yes	44	201	31.4	22.9	39.9
	Total	140	641	100.0		
Other Sites:Female Workers	No	50	463	65.0	53.2	76.8
	Yes	25	250	35.0	23.2	46.8
	Total	75	713	100.0		
Other Sites:Female Patrons	No	83	761	72.3	63.9	80.8
	Yes	30	291	27.7	19.2	36.1
	Total	113	1052	100.0		
Clubs Male Patrons	No	120	549	74.5	66.6	82.4
	Yes	41	188	25.5	17.6	33.4
	Total	161	737	100.0		
Other Sites:Male Patrons: run	No	84	793	74.1	67.2	81.1
	Yes	29	277	25.9	18.9	32.8
	Total	113	1070	100.0		
Total	No	707	4644			
	Yes	284	1888			
	Total	991	6531			

Table of Group by age24

Group	age24	Frequency	Weighted Frequency	Row Percent	95% Confidence Limits for Row Percent	
Street:Female Workers	No	127	953	69.0	58.3	79.8
	Yes	57	428	31.0	20.2	41.7
	Total	184	1380	100.0		
Clubs:Female Workers	No	120	549	58.5	50.0	67.1
	Yes	85	389	41.5	32.9	50.0
	Total	205	938	100.0		
Clubs Female Patrons	No	64	293	45.7	40.3	51.1
	Yes	76	348	54.3	48.9	59.7
	Total	140	641	100.0		

Other Sites:Female Workers	No	55	529	74.2	62.9	85.5
	Yes	20	184	25.8	14.5	37.1
	Total	75	713	100.0		
Other Sites:Female Patrons	No	69	645	61.3	49.7	72.9
	Yes	44	407	38.7	27.1	50.3
	Total	113	1052	100.0		
Clubs Male Patrons	No	109	499	67.7	60.9	74.5
	Yes	52	238	32.3	25.5	39.1
	Total	161	737	100.0		
Other Sites:Male Patrons: run	No	76	733	68.5	58.2	78.8
	Yes	37	337	31.5	21.2	41.8
	Total	113	1070	100.0		
Total	No	620	4200			
	Yes	371	2331			
	Total	991	6531			

Table of Group by loweduc

Group	loweduc	Frequency	Weighted Frequency	Row Percent	95% Confidence Limits for Row Percent	
Street:Female Workers	No	167	1253	90.8	86.8	94.8
	Yes	17	128	9.2	5.2	13.2
	Total	184	1380	100.0		
Clubs:Female Workers	No	198	906	96.6	93.4	99.8
	Yes	7	32	3.4	0.2	6.6
	Total	205	938	100.0		
Clubs Female Patrons	No	138	632	98.6	96.6	100.0
	Yes	2	9	1.4	0.0	3.4
	Total	140	641	100.0		
Other Sites:Female Workers	No	75	713	100.0	100.0	100.0
	Yes	0	.	.	.	.
	Total	75	713	100.0		
Other Sites:Female Patrons	No	106	982	93.3	88.6	98.0
	Yes	7	70	6.7	2.0	11.4
	Total	113	1052	100.0		
Clubs Male Patrons	No	157	719	97.5	94.6	100.0
	Yes	4	18	2.5	0.0	5.4
	Total	161	737	100.0		
Other Sites:Male Patrons: run	No	108	1017	95.0	90.8	99.2
	Yes	5	53	5.0	0.8	9.2
	Total	113	1070	100.0		
Total	No	949	6221			
	Yes	42	311			
	Total	991	6531			

Table of Group by unemployed

Group	unemployed	Frequency	Weighted Frequency	Row Percent	95% Confidence Limits for Row Percent	
Street:Female Workers	No	100	750	54.6	44.9	64.4
	Yes	83	623	45.4	35.6	55.1
	Total	183	1373	100.0		
Clubs:Female Workers	No	167	764	81.5	71.6	91.3

	Yes	38	174	18.5	8.7	28.4
	Total	205	938	100.0		
Clubs Female Patrons	No	80	366	57.1	49.5	64.8
	Yes	60	275	42.9	35.2	50.5
	Total	140	641	100.0		
Other Sites:Female Workers	No	71	680	95.3	89.7	100.0
	Yes	4	33	4.7	0.0	10.3
	Total	75	713	100.0		
Other Sites:Female Patrons	No	69	645	61.8	52.1	71.5
	Yes	43	399	38.2	28.5	47.9
	Total	112	1044	100.0		
Clubs Male Patrons	No	123	563	76.9	68.6	85.1
	Yes	37	169	23.1	14.9	31.4
	Total	160	732	100.0		
Other Sites:Male Patrons: run	No	90	861	80.5	73.1	87.9
	Yes	23	209	19.5	12.1	26.9
	Total	113	1070	100.0		
Total	No	700	4629			
	Yes	288	1882			
	Total	988	6511			

Frequency Missing = 3

Table of Group by lowses

Group	lowses	Frequency	Weighted Frequency	Row Percent	95% Confidence Limits for Row Percent	
Street:Female Workers	No	136	1020	73.9	63.9	83.9
	Yes	48	360	26.1	16.1	36.1
	Total	184	1380	100.0		
Clubs:Female Workers	No	151	691	73.7	61.8	85.5
	Yes	54	247	26.3	14.5	38.2
	Total	205	938	100.0		
Clubs Female Patrons	No	116	531	82.9	72.4	93.3
	Yes	24	110	17.1	6.7	27.6
	Total	140	641	100.0		
Other Sites:Female Workers	No	54	502	70.5	54.7	86.3
	Yes	21	210	29.5	13.7	45.3
	Total	75	713	100.0		
Other Sites:Female Patrons	No	94	877	83.3	73.6	93.0
	Yes	19	176	16.7	7.0	26.4
	Total	113	1052	100.0		
Clubs Male Patrons	No	120	549	74.5	62.5	86.6
	Yes	41	188	25.5	13.4	37.5
	Total	161	737	100.0		
Other Sites:Male Patrons: run	No	83	785	73.4	62.5	84.2
	Yes	30	285	26.6	15.8	37.5
	Total	113	1070	100.0		
Total	No	754	4955			
	Yes	237	1576			
	Total	991	6531			

Table of Group by lowliteracy

Group	lowliteracy	Frequency	Weighted Frequency	Row Percent	95% Confidence Limits for Row Percent	
Street:Female Workers	No	170	1275	92.4	87.5	97.3
	Yes	14	105	7.6	2.7	12.5
	Total	184	1380	100.0		
Clubs:Female Workers	No	202	925	98.5	97.0	100.0
	Yes	3	14	1.5	0.0	3.0
	Total	205	938	100.0		
Clubs Female Patrons	No	139	636	99.3	97.8	100.0
	Yes	1	5	0.7	0.0	2.2
	Total	140	641	100.0		
Other Sites:Female Workers	No	75	713	100.0	100.0	100.0
	Yes	0	.	.	.	.
	Total	75	713	100.0		
Other Sites:Female Patrons	No	112	1044	99.2	97.8	100.0
	Yes	1	8	0.8	0.0	2.2
	Total	113	1052	100.0		
Clubs Male Patrons	No	159	728	98.8	97.1	100.0
	Yes	2	9	1.2	0.0	2.9
	Total	161	737	100.0		
Other Sites:Male Patrons: run	No	105	992	92.7	85.8	99.5
	Yes	8	78	7.3	0.5	14.2
	Total	113	1070	100.0		
Total	No	962	6312			
	Yes	29	219			
	Total	991	6531			

Table of Group by jailed

Group	jailed	Frequency	Weighted Frequency	Row Percent	95% Confidence Limits for Row Percent	
Street:Female Workers	No	120	900	65.2	59.2	71.3
	Yes	64	480	34.8	28.7	40.8
	Total	184	1380	100.0		
Clubs:Female Workers	No	153	700	74.6	67.6	81.6
	Yes	52	238	25.4	18.4	32.4
	Total	205	938	100.0		
Clubs Female Patrons	No	113	517	80.7	75.1	86.4
	Yes	27	124	19.3	13.6	24.9
	Total	140	641	100.0		
Other Sites:Female Workers	No	68	655	91.8	85.8	97.9
	Yes	7	58	8.2	2.1	14.2
	Total	75	713	100.0		
Other Sites:Female Patrons	No	98	916	87.0	81.5	92.5
	Yes	15	137	13.0	7.5	18.5
	Total	113	1052	100.0		
Clubs Male Patrons	No	105	481	65.2	56.7	73.7
	Yes	56	256	34.8	26.3	43.3
	Total	161	737	100.0		
Other Sites:Male Patrons: run	No	78	744	69.5	59.7	79.2
	Yes	35	327	30.5	20.8	40.3
	Total	113	1070	100.0		

Total	No	735	4912
	Yes	256	1619
	Total	991	6531

Table of Group by raped

Group	raped	Frequency	Weighted Frequency	Row Percent	95% Confidence Limits for Row Percent	
Street:Female Workers	No	145	1088	78.8	72.5	85.1
	Yes	39	293	21.2	14.9	27.5
	Total	184	1380	100.0		
Clubs:Female Workers	No	159	728	77.6	72.7	82.4
	Yes	46	211	22.4	17.6	27.3
	Total	205	938	100.0		
Clubs Female Patrons	No	113	517	80.7	73.6	87.8
	Yes	27	124	19.3	12.2	26.4
	Total	140	641	100.0		
Other Sites:Female Workers	No	66	632	88.7	82.2	95.1
	Yes	9	81	11.3	4.9	17.8
	Total	75	713	100.0		
Other Sites:Female Patrons	No	105	980	93.1	88.8	97.4
	Yes	8	72	6.9	2.6	11.2
	Total	113	1052	100.0		
Clubs Male Patrons	No	154	705	95.7	92.4	98.9
	Yes	7	32	4.3	1.1	7.6
	Total	161	737	100.0		
Other Sites:Male Patrons: run	No	110	1039	97.1	93.8	100.0
	Yes	3	31	2.9	0.0	6.2
	Total	113	1070	100.0		
Total	No	852	5689			
	Yes	139	843			
	Total	991	6531			

Table of Group by homeless

Group	homeless	Frequency	Weighted Frequency	Row Percent	95% Confidence Limits for Row Percent	
Street:Female Workers	No	153	1148	83.2	77.6	88.7
	Yes	31	233	16.8	11.3	22.4
	Total	184	1380	100.0		
Clubs:Female Workers	No	174	796	84.9	80.0	89.7
	Yes	31	142	15.1	10.3	20.0
	Total	205	938	100.0		
Clubs Female Patrons	No	128	586	91.4	87.3	95.6
	Yes	12	55	8.6	4.4	12.7
	Total	140	641	100.0		
Other Sites:Female Workers	No	73	696	97.7	94.5	100.0
	Yes	2	17	2.3	0.0	5.5
	Total	75	713	100.0		
Other Sites:Female Patrons	No	107	997	94.7	90.5	98.9
	Yes	6	56	5.3	1.1	9.5
	Total	113	1052	100.0		
Clubs Male Patrons	No	132	604	82.0	77.1	86.9
	Yes	29	133	18.0	13.1	22.9

	Total	161	737	100.0		
Other Sites:Male Patrons: run	No	97	931	87.0	79.8	94.2
	Yes	16	139	13.0	5.8	20.2
	Total	113	1070	100.0		
Total	No	864	5758			
	Yes	127	773			
	Total	991	6531			

Table of Group by sexwork3mon

Group	sexwork3mon	Frequency	Weighted Frequency	Row Percent	95% Confidence Limits for Row Percent	
Street:Female Workers	No	18	135	9.8	1.8	17.8
	Yes	166	1245	90.2	82.2	98.2
	Total	184	1380	100.0		
Clubs:Female Workers	No	57	261	27.8	21.5	34.1
	Yes	148	677	72.2	65.9	78.5
	Total	205	938	100.0		
Clubs Female Patrons	No	97	444	69.3	61.5	77.1
	Yes	43	197	30.7	22.9	38.5
	Total	140	641	100.0		
Other Sites:Female Workers	No	73	696	97.7	94.4	100.0
	Yes	2	17	2.3	0.0	5.6
	Total	75	713	100.0		
Other Sites:Female Patrons	No	104	978	92.9	86.9	98.9
	Yes	9	75	7.1	1.1	13.1
	Total	113	1052	100.0		
Clubs Male Patrons	No	141	645	87.6	82.1	93.1
	Yes	20	92	12.4	6.9	17.9
	Total	161	737	100.0		
Other Sites:Male Patrons: run	No	107	1014	94.8	90.2	99.3
	Yes	6	56	5.2	0.7	9.8
	Total	113	1070	100.0		
Total	No	597	4173			
	Yes	394	2358			
	Total	991	6531			

Table of Group by paidcash

Group	paidcash	Frequency	Weighted Frequency	Row Percent	95% Confidence Limits for Row Percent	
Street:Female Workers	No	143	1073	78.6	71.0	86.2
	Yes	39	293	21.4	13.8	29.0
	Total	182	1365	100.0		
Clubs:Female Workers	No	181	828	89.2	85.1	93.2
	Yes	22	101	10.8	6.8	14.9
	Total	203	929	100.0		
Clubs Female Patrons	No	124	568	89.2	83.6	94.8
	Yes	15	69	10.8	5.2	16.4
	Total	139	636	100.0		
Other Sites:Female Workers	No	73	696	97.7	94.5	100.0
	Yes	2	17	2.3	0.0	5.5
	Total	75	713	100.0		

Other Sites:Female Patrons	No	108	993	95.6	90.0	100.0
	Yes	4	45	4.4	0.0	10.0
	Total	112	1038	100.0		
Clubs Male Patrons	No	86	394	53.4	44.8	62.0
	Yes	75	343	46.6	38.0	55.2
	Total	161	737	100.0		
Other Sites:Male Patrons: run	No	73	702	65.6	57.5	73.7
	Yes	40	368	34.4	26.3	42.5
	Total	113	1070	100.0		
Total	No	788	5253			
	Yes	197	1235			
	Total	985	6488			

Frequency Missing = 6

Table of Group by crack

Group	crack	Frequency	Weighted Frequency	Row Percent	95% Confidence Limits for Row Percent	
Street:Female Workers	No	181	1358	98.4	96.6	100.0
	Yes	3	23	1.6	0.0	3.4
	Total	184	1380	100.0		
Clubs:Female Workers	No	203	929	99.0	97.7	100.0
	Yes	2	9	1.0	0.0	2.3
	Total	205	938	100.0		
Clubs Female Patrons	No	140	641	100.0	100.0	100.0
	Yes	0	.	.	.	.
	Total	140	641	100.0		
Other Sites:Female Workers	No	75	713	100.0	100.0	100.0
	Yes	0	.	.	.	.
	Total	75	713	100.0		
Other Sites:Female Patrons	No	113	1052	100.0	100.0	100.0
	Yes	0	.	.	.	.
	Total	113	1052	100.0		
Clubs Male Patrons	No	160	732	99.4	98.2	100.0
	Yes	1	5	0.6	0.0	1.8
	Total	161	737	100.0		
Other Sites:Male Patrons: run	No	111	1048	97.9	94.9	100.0
	Yes	2	23	2.1	0.0	5.1
	Total	113	1070	100.0		
Total	No	983	6472			
	Yes	8	59			
	Total	991	6531			

Table of Group by dailyalc

Group	dailyalc	Frequency	Weighted Frequency	Row Percent	95% Confidence Limits for Row Percent	
Street:Female Workers	No	146	1095	79.3	73.3	85.4
	Yes	38	285	20.7	14.6	26.7
	Total	184	1380	100.0		
Clubs:Female Workers	No	127	581	62.0	54.6	69.3
	Yes	78	357	38.0	30.7	45.4
	Total	205	938	100.0		
Clubs Female Patrons	No	107	490	76.4	68.4	84.5

	Yes	33	151	23.6	15.5	31.6
	Total	140	641	100.0		
Other Sites:Female Workers	No	63	601	84.3	75.6	93.0
	Yes	12	112	15.7	7.0	24.4
	Total	75	713	100.0		
Other Sites:Female Patrons	No	100	944	89.7	82.8	96.7
	Yes	13	108	10.3	3.3	17.2
	Total	113	1052	100.0		
Clubs Male Patrons	No	114	522	70.8	63.9	77.7
	Yes	47	215	29.2	22.3	36.1
	Total	161	737	100.0		
Other Sites:Male Patrons: run	No	89	817	76.3	65.0	87.7
	Yes	24	253	23.7	12.3	35.0
	Total	113	1070	100.0		
Total	No	746	5050			
	Yes	245	1481			
	Total	991	6531			

Table of Group by venuefreq

Group	venuefreq	Frequency	Weighted Frequency	Row Percent	95% Confidence Limits for Row Percent	
Street:Female Workers	No	97	728	52.7	40.4	65.0
	Yes	87	653	47.3	35.0	59.6
	Total	184	1380	100.0		
Clubs:Female Workers	No	64	293	31.2	23.0	39.4
	Yes	141	645	68.8	60.6	77.0
	Total	205	938	100.0		
Clubs Female Patrons	No	111	508	79.3	70.8	87.8
	Yes	29	133	20.7	12.2	29.2
	Total	140	641	100.0		
Other Sites:Female Workers	No	12	112	15.7	5.4	25.9
	Yes	63	601	84.3	74.1	94.6
	Total	75	713	100.0		
Other Sites:Female Patrons	No	72	688	65.4	51.6	79.2
	Yes	41	365	34.6	20.8	48.4
	Total	113	1052	100.0		
Clubs Male Patrons	No	125	572	77.6	69.2	86.1
	Yes	36	165	22.4	13.9	30.8
	Total	161	737	100.0		
Other Sites:Male Patrons: run	No	73	690	64.5	52.3	76.6
	Yes	40	380	35.5	23.4	47.7
	Total	113	1070	100.0		
Total	No	554	3590			
	Yes	437	2941			
	Total	991	6531			

Table of Group by twoplaces

Group	twoplaces	Frequency	Weighted Frequency	Row Percent	95% Confidence Limits for Row Percent	
Street:Female Workers	No	118	885	64.1	54.4	73.8
	Yes	66	495	35.9	26.2	45.6

	Total	184	1380	100.0		
Clubs:Female Workers	No	160	732	78.0	73.2	82.9
	Yes	45	206	22.0	17.1	26.8
	Total	205	938	100.0		
Clubs Female Patrons	No	84	384	60.0	47.9	72.1
	Yes	56	256	40.0	27.9	52.1
	Total	140	641	100.0		
Other Sites:Female Workers	No	71	668	93.7	87.6	99.7
	Yes	4	45	6.3	0.3	12.4
	Total	75	713	100.0		
Other Sites:Female Patrons	No	71	674	64.0	53.7	74.3
	Yes	42	379	36.0	25.7	46.3
	Total	113	1052	100.0		
Clubs Male Patrons	No	100	458	62.1	53.4	70.9
	Yes	61	279	37.9	29.1	46.6
	Total	161	737	100.0		
Other Sites:Male Patrons: run	No	71	680	63.5	53.9	73.0
	Yes	42	391	36.5	27.0	46.1
	Total	113	1070	100.0		
Total	No	675	4480			
	Yes	316	2051			
	Total	991	6531			

Table of Group by nevertested

Group	nevertested	Frequency	Weighted Frequency	Row Percent	95% Confidence Limits for Row Percent	
Street:Female Workers	No	169	1268	91.8	86.8	96.9
	Yes	15	113	8.2	3.1	13.2
	Total	184	1380	100.0		
Clubs:Female Workers	No	192	879	93.7	91.1	96.3
	Yes	13	60	6.3	3.7	8.9
	Total	205	938	100.0		
Clubs Female Patrons	No	109	499	77.9	70.1	85.6
	Yes	31	142	22.1	14.4	29.9
	Total	140	641	100.0		
Other Sites:Female Workers	No	52	492	69.0	59.4	78.6
	Yes	23	221	31.0	21.4	40.6
	Total	75	713	100.0		
Other Sites:Female Patrons	No	81	763	72.5	65.2	79.7
	Yes	32	290	27.5	20.3	34.8
	Total	113	1052	100.0		
Clubs Male Patrons	No	91	417	56.5	47.4	65.7
	Yes	70	320	43.5	34.3	52.6
	Total	161	737	100.0		
Other Sites:Male Patrons: run	No	67	640	59.8	49.0	70.7
	Yes	46	430	40.2	29.3	51.0
	Total	113	1070	100.0		
Total	No	761	4956			
	Yes	230	1575			
	Total	991	6531			

Table of Group by tested12

Group	tested12	Frequency	Weighted Frequency	Row Percent	95% Confidence Limits for Row Percent	
Street:Female Workers	No	37	278	20.1	13.1	27.1
	Yes	147	1103	79.9	72.9	86.9
	Total	184	1380	100.0		
Clubs:Female Workers	No	49	224	23.9	15.5	32.3
	Yes	156	714	76.1	67.7	84.5
	Total	205	938	100.0		
Clubs Female Patrons	No	68	311	48.6	40.8	56.3
	Yes	72	330	51.4	43.7	59.2
	Total	140	641	100.0		
Other Sites:Female Workers	No	48	453	63.5	52.4	74.6
	Yes	27	260	36.5	25.4	47.6
	Total	75	713	100.0		
Other Sites:Female Patrons	No	66	638	60.6	50.7	70.5
	Yes	47	414	39.4	29.5	49.3
	Total	113	1052	100.0		
Clubs Male Patrons	No	109	499	67.7	59.5	75.9
	Yes	52	238	32.3	24.1	40.5
	Total	161	737	100.0		
Other Sites:Male Patrons: run	No	68	643	60.0	48.2	71.9
	Yes	45	428	40.0	28.1	51.8
	Total	113	1070	100.0		
Total	No	445	3045			
	Yes	546	3486			
	Total	991	6531			

Table of Group by Q51E

Group	Q51E	Frequency	Weighted Frequency	Row Percent	95% Confidence Limits for Row Percent	
Street:Female Workers	No	41	308	23.2	14.3	32.0
	Yes	136	1020	76.8	68.0	85.7
	Total	177	1328	100.0		
Clubs:Female Workers	No	77	352	37.7	29.7	45.8
	Yes	127	581	62.3	54.2	70.3
	Total	204	934	100.0		
Clubs Female Patrons	No	94	430	82.5	71.4	93.5
	Yes	20	92	17.5	6.5	28.6
	Total	114	522	100.0		
Other Sites:Female Workers	No	48	453	68.4	58.3	78.5
	Yes	23	209	31.6	21.5	41.7
	Total	71	662	100.0		
Other Sites:Female Patrons	No	75	719	78.5	68.5	88.4
	Yes	23	197	21.5	11.6	31.5
	Total	98	916	100.0		
Clubs Male Patrons	No	106	485	84.1	75.0	93.3
	Yes	20	92	15.9	6.7	25.0
	Total	126	577	100.0		
Other Sites:Male Patrons: run	No	75	731	85.7	77.0	94.3
	Yes	14	122	14.3	5.7	23.0
	Total	89	853	100.0		

Total	No	516	3477			
	Yes	363	2313			
	Total	879	5790			

Frequency Missing = 112

Table of Group by Q51F

Group	Q51F	Frequency	Weighted Frequency	Row Percent	95% Confidence Limits for Row Percent	
Street:Female Workers	No	37	278	20.8	13.2	28.4
	Yes	141	1058	79.2	71.6	86.8
	Total	178	1335	100.0		
Clubs:Female Workers	No	72	330	35.3	27.6	43.0
	Yes	132	604	64.7	57.0	72.4
	Total	204	934	100.0		
Clubs Female Patrons	No	92	421	80.7	70.3	91.1
	Yes	22	101	19.3	8.9	29.7
	Total	114	522	100.0		
Other Sites:Female Workers	No	49	461	69.7	59.7	79.6
	Yes	22	201	30.3	20.4	40.3
	Total	71	662	100.0		
Other Sites:Female Patrons	No	72	688	75.8	64.4	87.2
	Yes	25	220	24.2	12.8	35.6
	Total	97	907	100.0		
Clubs Male Patrons	No	104	476	82.5	73.0	92.1
	Yes	22	101	17.5	7.9	27.0
	Total	126	577	100.0		
Other Sites:Male Patrons: run	No	75	731	85.7	76.5	94.8
	Yes	14	122	14.3	5.2	23.5
	Total	89	853	100.0		
Total	No	501	3384			
	Yes	378	2406			
	Total	879	5789			

Frequency Missing = 112

Table of Group by Q51D

Group	Q51D	Frequency	Weighted Frequency	Row Percent	95% Confidence Limits for Row Percent	
Street:Female Workers	No	74	555	41.6	31.8	51.4
	Yes	104	780	58.4	48.6	68.2
	Total	178	1335	100.0		
Clubs:Female Workers	No	84	384	41.6	31.1	52.1
	Yes	118	540	58.4	47.9	68.9
	Total	202	925	100.0		
Clubs Female Patrons	No	93	426	67.4	58.0	76.8
	Yes	45	206	32.6	23.2	42.0
	Total	138	632	100.0		
Other Sites:Female Workers	No	44	425	61.1	48.2	74.0
	Yes	29	271	38.9	26.0	51.8
	Total	73	696	100.0		
Other Sites:Female Patrons	No	53	524	49.8	35.4	64.2
	Yes	60	528	50.2	35.8	64.6
	Total	113	1052	100.0		

Clubs Male Patrons	No	103	471	64.8	57.2	72.4
	Yes	56	256	35.2	27.6	42.8
	Total	159	728	100.0		
Other Sites:Male Patrons: run	No	74	687	64.6	57.3	72.0
	Yes	38	375	35.4	28.0	42.7
	Total	112	1062	100.0		
Total	No	525	3472			
	Yes	450	2957			
	Total	975	6429			

Frequency Missing = 16
